# Detecting Cortical Spreading Depolarization with Full Band Scalp Electroencephalography: An Illusion?

**DOI:** 10.3389/fneur.2018.00017

**Published:** 2018-01-25

**Authors:** Jeannette Hofmeijer, C. R. van Kaam, Babette van de Werff, Sarah E. Vermeer, Marleen C. Tjepkema-Cloostermans, Michel J. A. M. van Putten

**Affiliations:** ^1^Department of Clinical Neurophysiology, MIRA Institute for Biomedical Technology and Technical Medicine, University of Twente, Enschede, Netherlands; ^2^Department of Neurology, Rijnstate Hospital, Arnhem, Netherlands; ^3^Department of Clinical Neurophysiology, Medisch Spectrum Twente, Enschede, Netherlands

**Keywords:** cortical spreading depolarization, infraslow activity, acute ischemic stroke, traumatic brain injury, full band electroencephalography

## Abstract

**Introduction:**

There is strong evidence suggesting detrimental effects of cortical spreading depolarization (CSD) in patients with acute ischemic stroke and severe traumatic brain injury. Previous studies implicated scalp electroencephalography (EEG) features to be correlates of CSD based on retrospective analysis of EEG epochs after having detected “CSD” in time aligned electrocorticography. We studied the feasibility of CSD detection in a prospective cohort study with continuous EEG in 18 patients with acute ischemic stroke and 18 with acute severe traumatic brain injury.

**Methods:**

Full band EEG with 21 silver/silver chloride electrodes was started within 48 h since symptom onset. Five additional electrodes were used above the infarct. We visually analyzed all raw EEG data in epochs of 1 h. Inspection was directed at detection of the typical combination of CSD characteristics, i.e., (i) a large slow potential change (SPC) accompanied by a simultaneous amplitude depression of >1Hz activity, (ii) focal presentation, and (iii) spread reflected as appearance on neighboring electrodes with a delay.

**Results:**

In 3,035 one-hour EEG epochs, infraslow activity (ISA) was present in half to three quarters of the registration time. Typically, activity was intermittent with amplitudes of 40–220 µV, approximately half was oscillatory. There was no specific spatial distribution. Relevant changes of ISA were always visible in multiple electrodes, and not focal, as expected in CSD. ISA appearing as “SPC” was mostly associated with an amplitude increase of faster activities, and never with suppression. In all patients, depressions of spontaneous brain activity occurred. However, these were not accompanied by simultaneous SPC, occurred simultaneously on all channels, and were not focal, let alone spread, as expected in CSD.

**Conclusion:**

With full band scalp EEG in patients with cortical ischemic stroke or traumatic brain injury, we observed various ISA, probably modulating cortical excitability. However, we were unable to identify unambiguous characteristics of CSD.

## Introduction

Cortical spreading depolarization (CSD) is a wave of neuronal and glial depolarization, propagating across the brain’s gray matter at a velocity of 2–5 mm/min (1). CSD waves are associated with failure of brain ion homeostasis and lead to silencing of spontaneous and evoked synaptic transmission during 3 min under relatively healthy conditions to many hours in metabolically comprised tissue (2). The subsequent correction of ion gradients is accompanied with increased energy use (3, 4). CSD is a benign phenomenon in “healthy” brains with preserved perfusion and metabolism (5). As such, it plays a role in migraine aura, where the initial depolarization wave induces a wave of relative hyperperfusion called “spreading hyperemia” (6–9). Otherwise, in acutely injured brain tissue, with impaired cerebrovascular autoregulation and exhausted energy metabolism, CSD can induce spreading ischemia, which delays the neuronal repolarization and hence the recovery from the CSD-induced toxic intraneuronal changes, thereby increasing the risk of irreversible damage (10–13).

There is strong experimental and clinical evidence suggesting presence and potential detrimental effects of CSD in most, if not all, patients with acute cortical ischemic stroke, severe traumatic brain injury, and subarachnoid hemorrhage (10, 11, 14–21). With prevention of CSD being a possible treatment target, real time clinical measurement would be useful to guide eventual treatments. The Co-Operative Study on Brain Injury Depolarizations (COSBID) study group succeeded to detect CSD signatures with invasive subdural electrocorticography (ECoG) in patients with “malignant” ischemic stroke (14), traumatic brain injury (15), subarachnoid hemorrhage (22), and spontaneous intracerebral hemorrhage (10, 23). In these studies, CSD was defined as the combination of slow potential change (SPC) with simultaneous transient suppression of faster activities, propagating along the cortex. In the absence of any ECoG background activity (continuous depressions), which is a common situation in, for example, the ischemic penumbra, SPCs alone were designated as “silent” or “isoelectric” “peri-infarct depolarizations” (10).

For wide spread clinical application, reliable CSD detection should optimally be possible in a non-invasive way. The COSBID group implicated specific scalp electroencephalography (EEG) features to be correlates of CSD based on EEG studies combined with ECoG (24). Hereby, scalp EEG signatures were retrospectively sought for after having detected CSD in ECoG data. On EEG, SPC with a median amplitude of around −270 μV and duration of approximately 5.5 min, accompanied by suppression of faster activities, were adjudicated as CSD (24). In contrast to ECoG, only clusters of (and not isolated) CSD were identified, and EEG allowed no visualization of the typical spread. Still, it was concluded that (spreading) depolarizations and depressions of spontaneous activity in time-compressed human scalp EEG could serve as non-invasive signatures of spreading depolarizations and depressions.

We aim to study the feasibility of CSD detection, including its clinical relevance, by continuous full band scalp EEG in prospective cohort studies of patients with acute ischemic stroke and traumatic brain injury.

## Materials and Methods

### Design

We performed prospective cohort studies with continuous EEG monitoring for detection of CSD in patients with acute cortical ischemic stroke and acute traumatic brain injury. Patients with ischemic stroke were included at stroke units of two teaching hospitals. In Rijnstate Hospital, Arnhem, patients were included between February 2016 and October 2016; in Medisch Spectrum Twente, Enschede, between November 2014 and May 2015. Patients with acute traumatic brain injury were included at the intensive care unit of Medisch Spectrum Twente between November 2015 and December 2016.

### Approval and Consent

The Medical Research Ethics Committee Twente approved the research protocol for monitoring and follow-up of patients with ischemic stroke in October 2014 (registry number NL50284.044.14). Written and informed consent for continuous EEG monitoring and follow-up of patients with ischemic stroke was obtained from the patient or a legal representative, in case of a decreased consciousness or severe aphasia. Since continuous EEG monitoring and clinical follow-up of patients with a decreased consciousness are part of current care on the intensive care unit of MST, the Medical Research Ethics Committee Twente waived the need for informed consent in patients with acute traumatic brain injury.

### Patients

Inclusion criteria for patients with acute ischemic stroke were age ≥18 years, clinical symptoms consistent with a cortical localization, and severity leading to a score of ≥4 on the National Institutes of Health Stroke Scale (NIHSS). Exclusion criteria consisted of any progressive brain illness, expectation of short-term death due to stroke, and a pre-stroke modified Rankin Scale (mRS) score of >2. Treatment was according to standard protocols for patients with a brain infarct at the stroke unit, including early mobilization, if possible. Interference between the EEG recording and early mobilization was minimized by the use of small, mobile EEG equipment, if necessary. Inclusion criteria for patients with traumatic brain injury were admission to the intensive care unit with moderate to severe traumatic brain injury [Glasgow Coma Scale (GCS) ≤ 12] and age ≥18 years. Exclusion criteria were concomitant cardiac arrest or large open wounds, which hamper EEG registration. Treatment was according to standard protocols.

### Outcome

To relate eventual CSD candidates to clinical outcome, outcome measures were predefined and collected prospectively. The primary outcome measure for patients with ischemic stroke was functional outcome as expressed by the score on the mRS at 3 months after stroke. Secondary outcome measures included secondary deterioration during hospital admission, defined as an increase on the NIHSS score of two points or more. The NIHSS score was collected at the first presentation at the emergency room, at the start of EEG registration, every morning, at the end of EEG registration, and with any sign of neurological deterioration. The primary outcome measure for patients with traumatic brain injury was functional outcome as expressed by Extended Glasgow Outcome Scale at hospital discharge.

### EEG Registration

Electroencephalography registration started as soon as possible after admission to the stroke or intensive care unit, and within 48 h after symptom onset. For stroke patients, registration was aborted after 3 days, at discharge, or when the patient requested so, for example, in case of discomfort. In traumatic brain injury patients, registration was aborted after 7 days, at discharge, or when the patient regained consciousness. A Neurocenter EEG system (Clinical Science Systems, Leiden, the Netherlands) or (for mobile stroke patients) a portable Mobita EEG system (TMS International, Oldenzaal, the Netherlands) was used, with a DC-coupled amplifier (TMS International, Oldenzaal, the Netherlands, with the same settings for stationary and portable systems) and a sample frequency of 256 or 250 Hz. Twenty-one silver/silver chloride electrodes were placed on the scalp according to the international 10–20 system. In patients with ischemic stroke, five additional electrodes were placed above the infarcted area. These were placed on positions FC4, FC6, C6, CP6, and CP4 for right-sided, and FC3, FC5, C5, CP5, and CP3 for left sided infarcts. Electrodes were attached using collodion glue and filled with Ten20 conductive paste. Electrode impedance was controlled twice a day and kept below 5 kΩ. Recordings were obtained with the common reference.

### EEG Analysis

Signal processing was done with Matlab (MATLAB R2016a, The MathWorks Inc., Natick, MA, USA). Full band EEG recordings were stored in EDF+ format using the method described in Kemp et al. (25). Before further analysis, all EEGs were visually inspected in the frequency band of 1–35 Hz to assess the general quality of the recording. Epochs with loose electrodes and major artifacts were discarded.

Electroencephalography analysis was qualitative, by visual inspection. The search for CSD was done by inspection of all raw EEG data in epochs of 1 h. Data from patients with brain infarcts were inspected with bipolar electrode pairs, including four pairs including the additional electrodes above the infarcted hemisphere. Data from patients with traumatic brain injury, the Laplacian source derivation was used. For each electrode pair or electrode, the infraslow activity (ISA) (0.001–0.1 Hz), the “conventional” EEG activity (0.5–30 Hz), and the power of conventional activity were plotted. Stroke data were additionally inspected in the delta (1–4 Hz), theta (5–7 Hz), alpha (8–12 Hz), and beta (13–30 Hz) frequency bands. Filtering was achieved with a zero-phase second-order Butterworth bandpass filter (using the Matlab command “filtfilt”). Power was calculated every 30 s using a sliding window with the size of 60 s. The power spectral density was estimated using Welch’s method with a frequency resolution of 0.1 Hz, Hamming window, and 50% overlap (26). Because the ISA filter had a start-up effect, an extra 10 min preceding the 1 h section was always added and removed before visualization.

Visual inspection was directed at a qualitative description of ISA and detection of the typical combination of CSD characteristics. These consist of (i) a large SPC (=0.1 Hz) accompanied by a simultaneous amplitude depression of spontaneous >1 Hz activity, (ii) focal presentation of SPC and depression, and (iii) a spread of SPC and depression reflected as appearance on neighboring electrodes with a temporal delay. Sizes of the phenomena were based on previous studies, where the targeted SPC in the EEG had a median amplitude of 270–305 µV (range: 107–517) and a median duration of 5.5–7.4 min (IQR: 4.4–8.3) (24). As for depression of spontaneous brain activity, a median of 57% (IQR 44–67) and a duration of 21 min (median; IQR 16–33) have been reported (27).

## Results

### Patients

We included 18 patients with acute ischemic stroke, 11 in Rijnstate hospital, and 7 in MST. Baseline characteristics and outcomes are summarized in Table 1. The mean age was 72 (SD 11), and 44% was female. The median NIHSS on admission was 12 (IQR 7). All patients had an infarct in the middle cerebral artery (MCA) territory. EEG registration started after a median of 9 h after symptom onset (IQR 10). In five patients, worsening of neurological deficit was observed during EEG administration, four remained stable, and nine improved. In 13 stroke patients, registrations were discontinued because of discomfort. After 3 months, 14 patients had a poor outcome, of whom three had died. We included 18 patients with acute traumatic brain injury. Baseline characteristics and outcomes are summarized in Table 2. The mean age was 51 (SD 19), and 22% was female. The median GCS score on the site of trauma 7. EEG registration started after a median of 17 h after trauma. Six patients died on the intensive care unit.

**Table 1:** Baseline characteristics and outcome of patients with acute cortical ischemic stroke.

Patient	Age range (years)	Stroke location	EEG start (h after stroke)	Total EEG monitoring time (h)	NIHSS score admission	Deterioration time (h after stroke)	mRS 3 months
1	75–89	MCA R	1	22	11		4
2	70–74	MCA R	18	45	17		5
3	85–89	MCA L	17	42	18	22	5
4	50–54	MCA R	9	18	10	13	2
5	70–74	MCA R	6	63	16		3
6	70–74	MCA L	8	21	15	31	3
7	65–69	MCA L	5	17	8		2
8	65–69	MCA R	18	16	4	11	5
9	45–49	MCA L	15	2	21		2
10	70–74	MCA R	13	22	11		5
11	75–79	MCA L	18	20	12		4
12	80–84	MCA L	8	11	10		4
13	75–79	MCA R	6	20	15	6	6
14	85–89	MCA R	10	21	12		1
15	70–74	MCA L	7	26	20	26	6
16	85–89	MCA R	35	22	9		6
17	65–69	MCA L	7	44	10	23	5
18	50–54	MCA L	6	15	23		3

*M, male; F, female; MCA, middle cerebral artery; R, right; L, left; EEG, electroencephalography; NIHSS, National Institutes of Health Stroke Scale; mRS, modified Rankin Scale; mRS of 6, death*.

**Table 2:** Baseline characteristics and outcome of patients with acute traumatic brain injury.

Patient	Age range (years)	CT abnormalities	EEG start (h after trauma)	Total EEG monitoring time (h)	GCS at trauma site	GOSE at discharge
1	25–29	Bilateral	7	214	8	3
2	22–24	Bilateral and infratentorial	25	170	4	3
3	40–44	Left hemishere	17	139	7	2
4	70–74	Left hemishere	8	209	10	1
5	55–59	Bilateral	7	137	7	3
6	55–59	Bilateral and infratentorial	16	172	3	1
7	15–19	Left hemisphere and infratentorial	17	168	8	4
8	60–64	Bilateral	7	173	NA	4
9	20–24	Right hemisphere and infratentorial	130	45	3	4
10	40–44	Bilateral	21	97	3	4
11	75–79	Bilateral	14	45	6	2
12	15–19	Bilateral	32	144	NA	4
13	45–49	Bilateral	19	139	3	1
14	45–49	Bilateral	19	101	7	1
15	45–49	Bilateral and infratentorial	12	23	12	1
16	45–49	Bilateral	30	23	7	4
17	35–39	Bilateral and infratentorial	20	147	3	4
18	75–79	Bilateral and infratentorial	16	31	8	1

*M, male; F, female; CT, computed tomography; EEG, electroencephalography; GCS, Glasgow Coma Scale; GOSE, Extended Glasgow Outcome Scale; NA, not available*.

### Qualitative Description of ISA in Relation to “Conventional” EEG Activity

**Figure 1:**
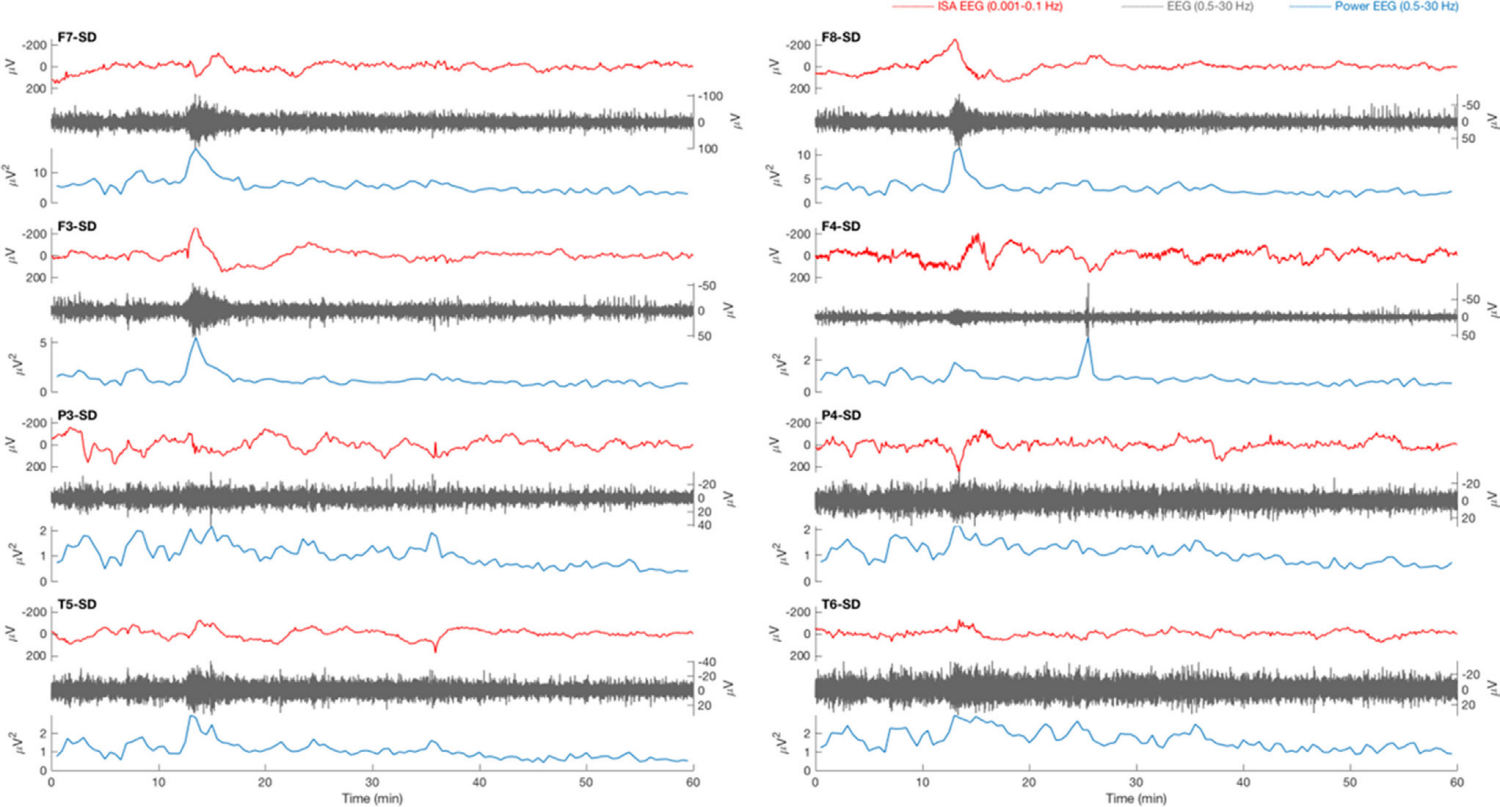
Typical example of a 1 h electroencephalography (EEG) overview from patient 18 with traumatic brain injury. Source derivation for four electrodes (F7, F3, P3, and T5) for the left hemisphere and four electrodes (F8, F4, P4, and T6) for the right hemisphere are displayed. The infraslow activity (ISA, 0.001–0.1 Hz, red) shows a slow potential change (SPC) at minute 13 on positions F7, F3, F8, F4, and P4. On other positions, the activity is less prominent. A simultaneous increase of the amplitude (gray) and power (blue) of faster EEG activity (0.5–30 Hz, gray) can be seen in all electrodes. The SPC is not focal, not accompanied by a depression of spontaneous activity, and there is no delay between appearances on the various electrodes, as expected in cortical spreading depolarization (CSD).

**Figure 2:**
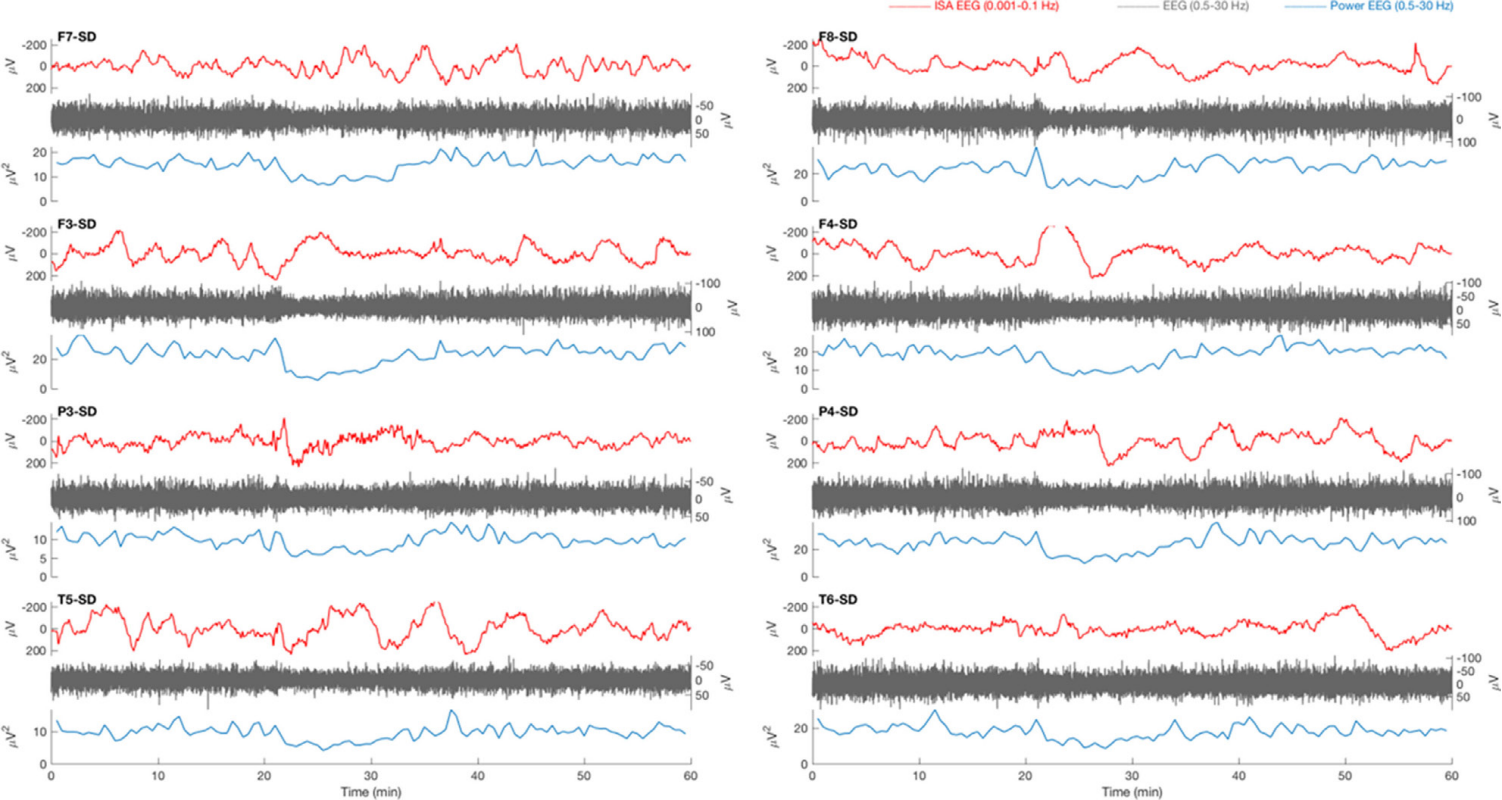
Typical example of a 1 h electroencephalography (EEG) overview from patient 8 with traumatic brain injury. Source derivations for four electrodes (F7, F3, P3, and T5) of the left hemisphere and four electrodes (F8, F4, P4, and T6) of the right hemisphere are displayed. At 20 min, in the infraslow frequency (ISA) band (0.001–0.1 Hz, red), a slow potential change (SPC) is present on all electrode positions except F7 and T6. The SPC is accompanied by a depression of spontaneous activity (0.5–30 Hz, gray), which is clearly visible in the power of this frequency band (blue). The suppression is not focal, as expected in cortical spreading depolarization (CSD), but occurs on all positions simultaneously.

**Figure 3:**
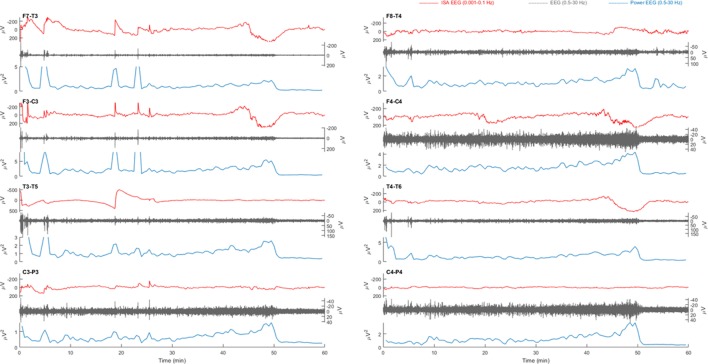
Example of a 1 h electroencephalography (EEG) overview from patient 3 with a left sided middle cerebral artery infarction. Electrode pairs F7-T3, F3-C3, T3-T5, and C3-P3 over the left hemisphere and F8-T4, F4-C4, T4-T6, and C4-P4 over the right hemisphere are displayed. In F7-T3, a possible slow potential change (SPC) is visible in the infraslow activity (ISA) band (0.001–0.1 Hz, red) at, e.g., 18 min. During this activity, there is an increase of faster activity (0.5–30 Hz, gray). On all electrode pairs, a sudden drop power of fast activities (blue) is present at 50 min. However, there is neither clear simultaneous SPC nor a unilateral presentation with spreading, as would be expected in cortical spreading depolarization (CSD).

We inspected 429 one-hour EEG epochs and an additional 429 overviews of extra electrodes above the infarcted area of the patients with infarcts. Approximately 40% of the epochs appeared to contain too may artifacts in the ISA band to further analyze. From patients with traumatic brain injury, 2,177 one-hour EEG epochs were inspected. A quarter contained too may artifacts in the ISA band to further analyze. In the remaining samples of both patient groups, ISA was present in half to three quarters of the registration time. Typically, activity was intermittent, where several minutes with abundant ISA were interspersed with minutes without ISA. Amplitudes were between 40 and 220 µV and frequencies from the complete ISA spectrum were observed. Although the predominance of ISA varied between electrodes, there was no specific spatial distribution. ISA was often associated with simultaneous amplitude fluctuations of faster activities (Figures 1–3).

**Figure 4:**
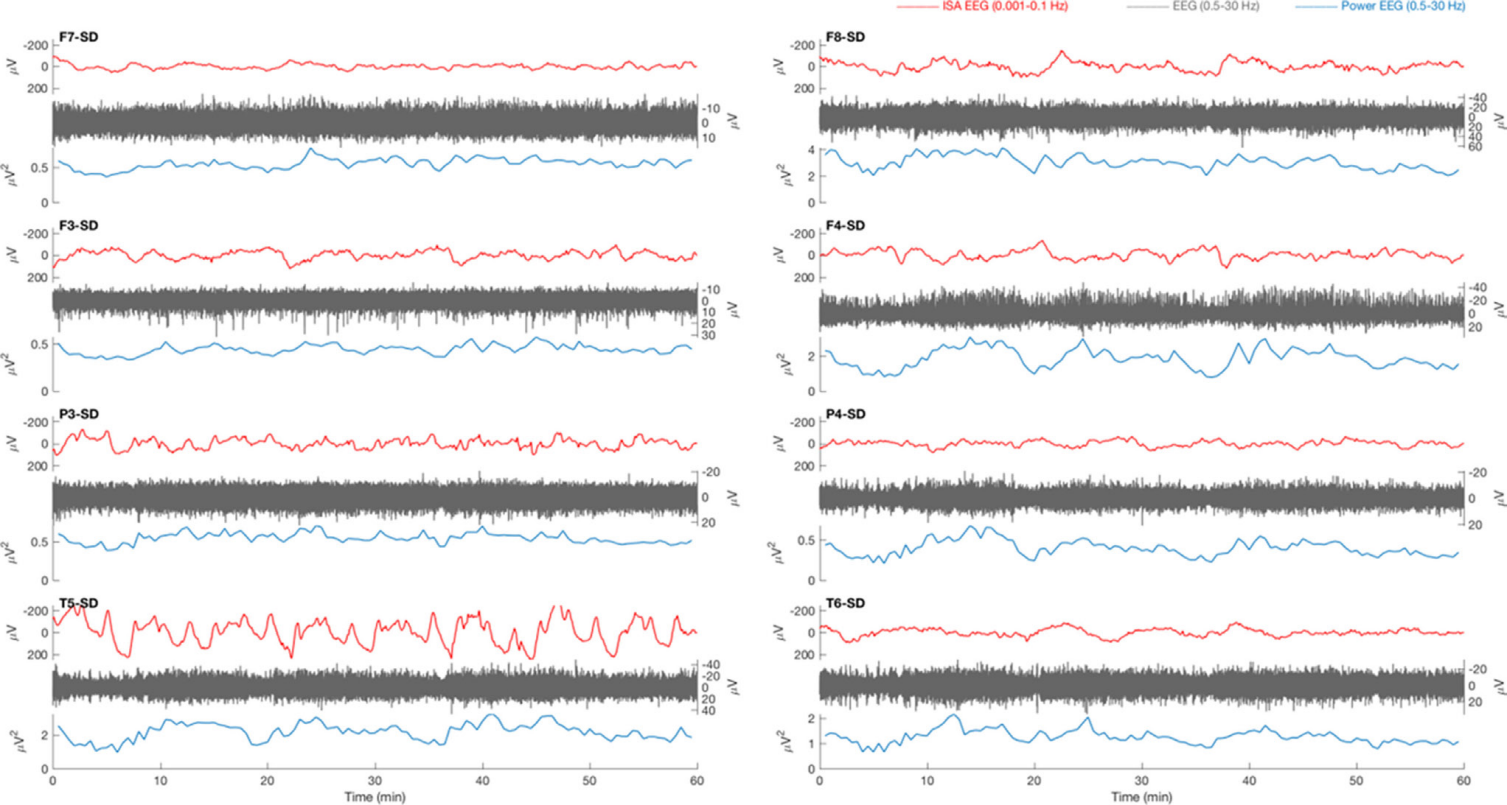
Example of a 1 h electroencephalography (EEG) overview from a patient with traumatic brain injury. Source derivations for four electrodes (F7, F3, P3, and T5) of the left hemisphere and four electrodes (F8, F4, P4, and T6) of the right hemisphere are displayed. Oscillatory infraslow activity (ISA, 0.001–0.1 Hz, red) is present over the right hemisphere. The ISA is accompanied by simultaneous fluctuations of faster activity (0.5–30 Hz, gray), which is visible in the power of this frequency band (blue). The fluctuations of faster activity result from waxing and waning of lateralized periodic discharges.

**Figure 5:**
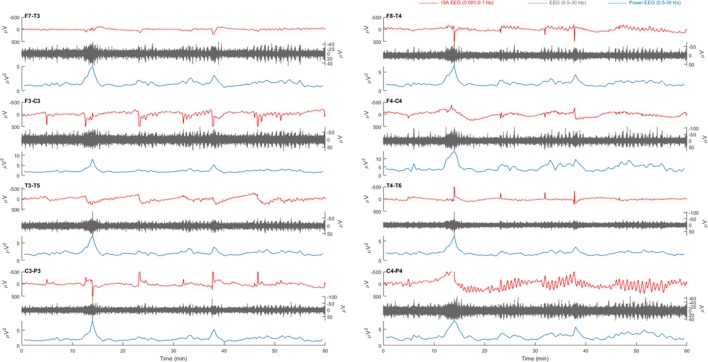
Example of a 1 h electroencephalography (EEG) overview from patient 1 with right-sided middle cerebral artery infarction. Electrode pairs F7-T3, F3-C3, T3-T5, and C3-P3 over the left hemisphere and F8-T4, F4-C4, T4-T6, and C4-P4 over the right hemisphere are displayed. Oscillatory infraslow activity (ISA, 0.001–0.1 Hz, red) is present over electrode pair C4-P4. The ISA is accompanied by simultaneous fluctuations of faster activity (0.5–30 Hz, gray), which is visible in the power of this frequency band (blue).

In five patients with brain infarcts and all patients with traumatic brain injury, periods of high amplitude (>100 μV) oscillatory ISA was observed. The duration of this oscillatory activity varied from 30 min to many hours. Frequencies typically ranged from 0.02 to 0.08 Hz. Phase–amplitude coupling was often seen to some extent, both in stroke and in trauma patients. In general, increased ISA periodicity was associated with increased phase–amplitude coupling (Figures 4 and 5). With increased ISA periodicity, we also observed an increase of pathological delta activity.

As expected, asymmetry was present in patients with hemispheric brain infarcts. In the conventional frequency band, this was mainly due to increased pathological delta power in the lesioned hemisphere. ISA amplitude, frequency and periodicity distributions appeared to be essentially the same in both hemispheres.

### No Successful CSD Detection

Relevant changes of ISA were always visible in multiple electrodes, simultaneously. Often, ISA appeared as “sharp negative spikes” on all positions and was accompanied by an increase of delta power. This activity was always seen in both hemispheres, simultaneously (Figures 1 and 3). There were no focal SPC-like phenomena, or SPCs that were accompanied by suppression of faster brain activity, as expected in CSD.

In all patients, drops of high amplitude delta activity were observed (Figure 3). However, these depressions of spontaneous brain activity were not accompanied by simultaneous SPC-like phenomena. Moreover, these always occurred simultaneously on many channels and were not focal, let alone spread, as expected in CSD. In sum, no CSD candidate was identified.

## Discussion

We were unable to identify unambiguous characteristics of CSD in patients with cortical ischemic stroke or traumatic brain injury, despite careful inspection of 3,035 h of continuous full band EEG in the infraslow and conventional frequency bands. This indicates that prospective CSD detection by full band scalp EEG is difficult. Of note, the inability to detect CSD does not undermine our belief in its existence and relevance.

Previous researchers have implicated identification of EEG signatures of CSD in patients with subarachnoid hemorrhage (24), malignant MCA infarction (24), or traumatic brain injury (27). Identification of CSD was always based on simultaneous recordings with subdural ECoG electrodes in patients that had been subject to craniotomy. After having identified depolarizations in ECoG recordings, scalp EEG correlates were examined by retrospective review of time aligned data. Unsupported by ECoG, in patients without craniotomy, we were unable to detect possible CSD with EEG. Factors that may be associated with this inability include artifacts, limited spatial resolution, inadequate visual analysis, and true inability to detect CSD with scalp EEG alone. These are discussed below.

By visual inspection of the complete, unfiltered EEG datasets, we observed various artifacts, including movement- and nursing procedure artifacts, contact phenomena, and changes of the redox potential at the electrode–skin interface. Artifacts influenced the infraslow components of the EEG more than the conventional frequency bands. Moreover, in lower frequency bands, EEG recovery from artifacts was slower. Especially in freely moving patients at the stroke unit, abundant artifacts in the ISA frequency bands hampered proper visual analysis. Artifacts in the ISA band are especially relevant for detection of isoelectric spreading depolarizations, i.e., spreading depolarizations in brain areas where conventional EEG activity is already depressed. In these cases, detection of spreading depolarization completely depends on detection of an SPC (24).

We used 21 conventional electrode positions with some additional electrodes above the assumed penumbra in patients with brain infarcts. The COSBID group EEG data on CSD were obtained with the same electrode configuration. Unlike in their ECoG data, in their EEG data, spread of amplitude depression was noticed in only a minority of patients with traumatic brain injury (27), and not in patients with brain infarcts (24). This may result from superposition of volume conducted EEG signals from widespread cortical generators (24). Otherwise, it may indicate that spatial resolution of a conventional EEG set up is insufficient to detect CSD, and higher density arrays are needed.

Even if we waive the criterion of spread, we were unable to identify candidate phenomena, since we did not observe the combination of clear SPC and simultaneous, focal amplitude depression of faster activity. Contrary, most features resembling SPC were accompanied by amplitude *increases* of faster activities. ISA associated with faster neuronal activities has been recognized since the late 1950s (28). There are roughly two views on the pathophysiology of this association. First, these could be an emergent property of the fast oscillations and thus be completely generated cortically. Second, these may result from distant mechanisms influencing cortical membrane potentials. Since CSD is a cortical phenomenon, CSD-associated SPCs should be generated cortically. This possibility is supported by the notion that negative potential shifts *can* arise as byproducts of fast activities (29). However, there is far more evidence supporting distant ISA generators, mostly subcortically, influencing cortical rhythms by modulation of cortical excitability (29). It is not possible to differentiate between the two mechanisms from ECoG or EEG measurements alone. Moreover, in higher mammals such as cats and monkeys, and hence also likely in humans, there may be a complex relationship between the scalp direct current potential and cerebral blood flow changes (30) or changes in partial pressures of CO_2_ or O_2_ (31, 32) at the blood–brain barrier. Therefore, and since the criterion of spread was not fulfilled in any of our and most of the COSBID measurements, in the absence of a validated gold standard for CSD detection we doubt the true nature of all reported phenomena.

Our study obviously has limitations. First, we analyzed all EEG data by visual inspection. We chose to do so, because visual inspection still is considered gold standard for EEG interpretation. This even applies to artifact detection algorithms, which have been validated against visual analyses, once. We assumed that, for clinically relevant EEG-based CSD detection, it should be possible to visually identify at least some CSD signatures. Our methods and findings do not exclude subtle effects in the EEG, to be identified by advanced signal processing. However, the nature of such effects, if identified, would remain uncertain and preclude use at the bedside. We therefore believe that our findings call into question the possibility of clinically relevant CSD detection by scalp EEG. Second, interpretation of ISA, oscillatory or not, was hampered by lack of data from a healthy population. Third, in trauma patients, effects of sedative medication are not taken into account.

In conclusion, with full band scalp EEG in patients with cortical ischemic stroke and traumatic brain injury, we observed various ISA, probably modulating cortical excitability. However, we were unable to identify unambiguous characteristics of CSD. We support the recently published consensus statement that invasive recordings are needed for detection of CSD and, so far, scalp EEG alone is not sufficient (33).

## Ethics Statement

The Medical Research Ethics Committee Twente approved the research protocol for monitoring and follow-up of patients with ischemic stroke in October 2014 (registry number NL50284.044.14). Informed consent for continuous EEG monitoring and follow-up patients with ischemic stroke was obtained from the patient or a legal representative, in case of a decreased consciousness or severe aphasia. Since continuous EEG monitoring of patients with a decreased consciousness is part of current care on the intensive care unit of MST, the Medical Research Ethics Committee Twente waived the need for informed consent in patients with acute traumatic brain injury.

## Author Contributions

JH: study design and conceptualization, data interpretation, and writing first draft; CK and BW: data collection, data analysis, and interpretation and revising the manuscript for intellectual content; SV: data collection and revising the manuscript for intellectual content; MT-C and MP: study design and conceptualization and data interpretation. All the authors: final approval and agreement to be accountable.

## Conflict of Interest Statement

MP is cofounder of Clinical Science Systems. The other authors report to have no conflicts of interest.

## Abbreviations

COSBID, Co-Operative Study on Brain Injury Depolarizations; CSD, cortical spreading depolarization; ECoG, electrocorticography; ISA, infraslow activity (0.001–0.1 Hz); mRS, modified Rankin Scale; NIHSS, National Institutes of Health Stroke Scale.
